# *mNanog* Possesses Dorsal Mesoderm-Inducing Ability by Modulating Both BMP and Activin/Nodal Signaling in *Xenopus* Ectodermal Cells

**DOI:** 10.1371/journal.pone.0046630

**Published:** 2012-10-11

**Authors:** Aya Miyazaki, Kentaro Ishii, Satoshi Yamashita, Susumu Nejigane, Shinya Matsukawa, Yuzuru Ito, Yasuko Onuma, Makoto Asashima, Tatsuo Michiue

**Affiliations:** 1 Department of Life Sciences (Biology), Graduate School of Arts and Sciences, the University of Tokyo, Tokyo, Japan; 2 Research Center for Stem Cell Engineering, National Institute of Advanced Industrial Science and Technology (AIST), Tsukuba City, Ibaraki, Japan; University of Colorado, Boulder, United States of America

## Abstract

**Background:**

In *Xenopus* early embryogenesis, various genes are involved with mesoderm formation. In particular, dorsal mesoderm contains the organizer region and induces neural tissues through the inhibition of bone morphogenetic protein (BMP) signaling. In our initial study to identify novel genes necessary for maintaining the undifferentiated state, we unexpectedly revealed mesoderm-inducing activity for *mNanog* in *Xenopus*.

**Methodology/Principal Findings:**

The present series of experiments investigated the effect of *mNanog* gene expression on *Xenopus* embryo. Ectopic expression of *mNanog* induced dorsal mesoderm gene activity, secondary axis formation, and weakly upregulated Activin/nodal signaling. The injection of *mNanog* also effectively inhibited the target genes of BMP signaling, while *Xvent2* injection downregulated the dorsal mesoderm gene expression induced by *mNanog* injection.

**Conclusions/Significance:**

These results suggested that *mNanog* expression induces dorsal mesoderm by regulating both Activin/nodal signaling and BMP signaling in *Xenopus*. This finding highlights the possibly novel function for *mNanog* in stimulating the endogenous gene network in *Xenopus* mesoderm formation.

## Introduction

Germ layer formation is one of the most important processes in the fundamental patterning of an embryo. In *Xenopus* early embryogenesis, mesoderm is induced by signals secreted from endodermal tissue during the blastula stage, and *nodal-related* (*Xnr*) genes are known to play important roles in this biological process. *VegT* and Wnt signaling induces ¥¥*nr5/6*, followed by the sequential upregulation of *Xnr1/2* and *Xnr4* [1]–[3], and consequently, various mesoderm gene activities. Activin A, a TGF-ß superfamily member, was first identified as a factor that could induce both ventral and dorsal mesoderm [4]. In dorsal mesoderm, also called the Spemann-Mangold organizer, several genes including *chordin* (*chd*), *noggin* (*nog*), *goosecoid* (*gsc*), and *xlim-1* are expressed to induce neural tissues in the presumptive neuroectoderm [5]–[8].

*Xenopus* blastula ectodermal cells, or animal cap (AC) cells, possess multipotency and can differentiate into many types of tissues including mesoderm. However, the period for mesoderm induction in AC is limited until early gastrula. This phenomenon is known as “loss of mesodermal competence" (LMC) [9]. To identify novel factors involved with maintaining multipotency in *Xenopus* embryo, we first attempted to find genes involved in releasing LMC.

The first candidate gene we examined was *mNanog*, which encodes a homeodomain protein and is efficiently expressed in mammalian embryonic stem (ES)/induced pluripotent stem (iPS) cells [10]–[12]. Our preliminary experiments revealed that in the presence of Activin A treatment, *mNanog* injection promotes AC elongation and some mesodermal gene expression even at the late gastrula stage (data not shown). We also unexpectedly found that m*Nanog* injection induces AC elongation without Activin A treatment and could promote the expression of dorsal mesoderm genes such as *chd*, *gsc*, and *xlim-1* in AC. Further experiments revealed showed that *mNanog* also weakly promotes Activin/nodal signaling and inhibits BMP signaling. Together, these data indicated that *mNanog* modulates both these signaling pathways to induce the dorsal mesoderm cell fate in *Xenopus* AC, suggesting a novel function for *mNanog* in embryogenesis.

## Materials and Methods

### Plasmids

The *mNanog* gene was amplified by RT-PCR with mouse cDNA (from mouse ES D3 cell line (American Type Culture Collection(ATCC)). All experiments with the mouse ES cells were approved by the institutional ethics committee (Graduate Schools of Arts and Sciences, University of Tokyo: #19-19 and #23-10). mNanog/SK was made by inserting the amplified fragment of *mNanog* into the *EcoR*V site of pBluescriptII SK-. For injection, we inserted the *EcoR*I-*Xho*I fragment of mNanog/SK into the *EcoR*I-*Xho*I site of pCS2 to construct mNanog/CS2. dnALK4/CS2, Xnr2/CS2, Xnr5/CS2, cmXnr1/CS2, cmXnr2/CS2, and Xvent2/CS2 were also used for microinjection [3], [13]–[16]. For lineage tracing, we used pCS2-*lacZ*.

### Microinjection

Microinjecion was performed using a picojector (Harvard Medical Instruments). RNA for injection was synthesized with the mMESSAGE mMACHINE SP6 kit (Ambion/Applied Biosystems). Injected embryo was obtained by artificial fertilization and dejellied with 4.6% L-cysteine hydrochloride solution. Injection was performed in 5% Ficoll/1 X Steinberg's Solution (SS). Injected embryos were cultured in 0.1 X SS solution. *Xenopus* maintenance was carried out in compliance with institutional regulations and all *Xenopus* experiments were approved by the institutional ethics committee noted above (#21-10 and #24-8).

### Animal cap assay

mRNA was injected into the animal pole region of 2-cell-stage embryos. ACs were dissected at the late blastula stage (Stage 9), and then cultured to the appropriate stage with/without treatment with 10 ng/ml of Activin A. The shape of treated ACs was observed at about 12 hours after treatment. Treated AC was also assessed by the expressions of several marker genes.

### RT-PCR

We synthesized cDNA with 0.3 µg of total RNA prepared from 5–10 ACs. For reverse transcription, we used Superscript III (Invitrogen), and PCR was carried out with Ex Taq DNA polymerase (Takara, Japan). Primer sets used for PCR were as follows:

ODC: GCCATTGTGAAGACTCTCTCCATTC and TTCGGGTGATTCCTTGCCAC; *Xbra*: AGCCTGTCTGTCAATGCTCC and ACTGAGACACTGGTGTGATGG;

*Chd*: AACTGCCAGGACTGGATGGT and GGCAGGATTTAGAGTTGCTTC;

*Gsc*: CACACAAAGTCGCAGAGTCTC and GGAGAGCAGAAGTTGGGGCCA; *Siamois*: TACCGCACTGACTCTGCAAG and CTGAGGCTCCTGTGGAATTC;

*Xnr1*: GCAGTTAATGATTTTACTGGC and CAACAAAGCCAAGGCATAAC;

*Xnr2*: ATCTGATGCCGTTCTAAGCC and GACCTTCTTCAACCTCAGCC;

*Xnr3*: CTTCTGCACTAGATTCTG and CAGCTTCTGGCCAAGACT;

*Xnr5*: TCACAATCCTTTCACTAGGGC and GGAACCTCTGAAAGGAAGGC;

*Xnr6*: TCCAGTATGATCCATCTGTTGC and TTCTCGTTCCTCTTGTGCCTT.

*Xvent1*: AAGTATGCCAAGGAGATGCC and AGCTTCTTCCGTTCAGATGC;

*Xvent2*: TGAGACTTGGGCACTGTCTG and CCTCTGTTGAATGGCTTGCT;

*Xwnt8*: AGATGACGGCATTCCAGA and TCTCCCGATATCTCAGGA;

*mix*: GTGTCACTGACACCAGAA and AATGTCTCAAGGCAGAGG;

*mixer*: CAATGTCACATCAACTGAAG and CACCAGCCCAGCACTTAACC;

*xlim-1*: CCCATCTAGTGACGCTCAGAGG and CCACACTGCCGTTTCGTTC;

*Cer*: CCACAGAATACAAGCCATGG and AGCTTCACACGTGCATTCC;

*mNanog*: GGCCCTGAGGAGGAGGAGAAC and TGCAAGCGGTGGCAGAAAAAC;

*EF1α*: CAGATTGGTGCTGGATATGC and ACTGCCTTGATGACTCCTAG;

*BMP4*: TTTCCCTTGGCTGATCACCTAAAC and TCAACGGCACCCACACCC.

*Xnot*: ATA CATGGTTGGCACTGA and CTCCTACAGTTCCACATC.

*ms-actin*: GCTGACAGAATGCAGAAG and TTGCTTGGAGGAGTGTGT.

*NCAM*: CACAGTTCCACCAAATGC and GGAATCAAGCGGTACAGA.

*Xnrp-1*: GGGTTTCTTGGAACAAGC and ACTGTGCAGGAACACAAG.

### *In situ* hybridization

Embryos were bleached in hydrogen peroxide-methanol before fixation in MEMFA (formaldehyde-MOPS solution) and dehydration with ethanol. Rehydrated embryos were hybridized with DIG-labeled probe for 24 h at 60°C. Embryos were then incubated with 2000× anti-DIG antibody (Roche) for 12 h, washed 5 times, and then visualized by reaction in NBT/BCIP solution (Roche).

### TUNEL staining

*In situ* TUNEL assay for detecting apoptotic cells were carried out by previous method [17]. Briefly, fixed and bleached embryos were incubated with TdT enzyme (Invitrogen) and DIG-dUTP (Roche) for 1day. After washing, embryos were incubated with anti-DIG antibody, washed with MAB and detected with BM-purple (Roche).

### Cycloheximide (CHX) treatment

The procedure for CHX treatment was basically carried out as previously described [18]. Normal or Injected embryos were treated with 40 ng/ml of CHX in 1× Steinberg's solution at Stage 7, and was homogenized at Stage 9.

### *Xenopus Nanog* gene cloning

To clone the *Xenopus* homolog of *Nanog* gene, we carried out degenerated PCR with following primers:

U1: CC(T/C)GA(T/C)TC(A/T)GCCACCAG(T/C)CC(A/C)AA(G/A),

U2: TC(A/T)CC(T/C)GA(T/C)TC(A/T)GCCACCAG(T/C)CC(A/C),

L1: CTGGAACCAG(G/T)TCTT(A/C)ACCTG,

L2: CAT(T/C)CT(A/T)CG(G/A)TTCTGGAACCA,

L3: TTCAT(T/C)CT(A/T)CG(G/A)TTCTGGAACCAG, and

L4: G(G/T)TCTT(A/C)ACCTG(T/C)TTGTA(G/T)GTGAG.

The positions of these primers are summarized in Fig. S1.

## Results

### *mNanog* injection stimulated mesoderm-inducing activity in AC

**Figure 1:**
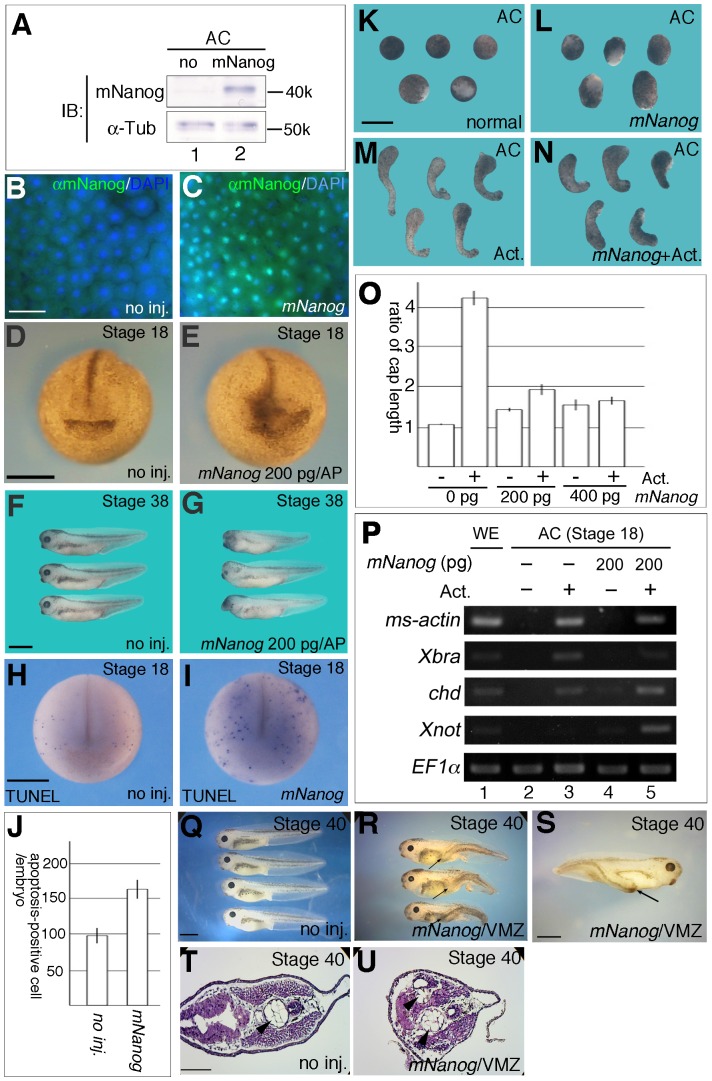
Mesodermal tissues were induced by *mNanog* mRNA injection. A) Detection of exogenous mNanog protein in *Xenopus* embryo by Western blotting with antibodies to mNanog (upper) and alpha-tubulin (lower). Non-injected embryo control (lane 1). *mNanog*-injected embryo (lane 2). B–C) Subcellular localization of mNanog protein in stage-10 embryo injected with *mNanog* mRNA. Ectoderm from normal embryo (B) or *mNanog*-injected embryo (C). Dissected tissues were fixed at stage 9, and then treated with anti-mNanog antibody. The green signal indicates mNanog protein. DAPI staining was also done (blue). Scale bar; 0.02 mm. D-G) Superficial phenotype of *mNanog*-injected embryos. Stage-18 (D, E) and stage-38 embryos (F, G) were observed. (D, F) Uninjected embryo. (E, G) 200 pg of *mNanog* mRNA was microinjected into the animal pole region at the 4-cell stage. Scale bar; 0.5 mm (D) and 1 mm (F). H, I) TUNEL staining of normal (H) or *mNanog*-injected (200 pg: I) embryos was performed at stage 20. Apoptotic cells appear as blue dots. J) The number of apoptosis-positive cells in normal embryo (n = 14) and 200 pg of *mNanog* injected embryo (n = 18) was described in bar graph. Error bar indicates S.E. K–N) Comparison of AC shapes between *mNanog*-injected embryos with and without Activin A treatment. All ACs were dissected at stage 9 and observed at stage 18. Normal ACs (K). ACs injected with *mNanog* into the animal pole region (L). ACs treated with 10 ng/ml Activin A at stage 9 (M). *mNanog*-injected ACs treated with Activin A at stage 9 (N). Scale bar; 0.5 mm. O) Analysis of AC elongation in (K)–(N). Both the shortest and longest lengths of AC were measured, and averages of the length ratio (long/short) were expressed as bar graphs. Normal AC (n = 50), 10 ng/ml Activin A-treated AC (n = 58), AC with 200 pg of *mNanog* injected (n = 46), AC with 200 pg of *mNanog* injected and 10 ng/ml Activin A treatment (n = 47), 400 pg of *mNanog* injected AC (n = 52), 400 pg of *mNanog* injected and 10 ng/ml Activin A treatment (n = 42). Error bar indicates S.E. P) RT-PCR analysis with RNA derived from stage-18 AC. Normal AC (lane 2), AC with 10 ng/ml of Activin A treatment (lane 3), AC injected with 200 pg of *mNanog* (lane 4), or AC injected with 200 pg of *mNanog* and treated with Activin A (lane 5) were used. WE: whole embryo. Q–S) Secondary axis formation with *mNanog* injection. 400 pg of *mNanog* mRNA was injected into the ventral marginal zone (VMZ) at the 4-cell stage. Phenotypes were observed at stage 40. Secondary axis without head structure was observed in *mNanog*-injected embryo (15/30, two independent experiments). Arrow indicates a secondary axis. Scale bar; 1 mm. T, U) HE-stained histological sections of stage-40 embryo. Uninjected embryo (T). An embryo injected with 200 pg of *mNanog* mRNA into the VMZ (U). Arrowhead indicates notochord-like structure. Scale bar: 0.2 mm.

At first, we confirmed the expression of mNanog protein in *Xenopus* embryo. By Western blot analysis, we could detect a protein of 40 kDa, consistent with the molecular size of the mNanog protein (Fig. 1A). Immunohistochemistry with anti-mNanog antibody showed intense mNanog reactivity in the nuclei of *mNanog*-injected embryos (Fig. 1B, C). Next, we examined the effects of *mNanog* on *Xenopus* early embryogenesis. 200 pg of m*Nanog* mRNA injected into the animal pole of 4-cell embryos caused a defect in the anterior region at the late neural stage (Fig. 1D, E), although no obvious developmental delay was observed (data not shown). In 3-day-old tadpoles, head defects with small eye vesicles could be seen (Fig. 1G, Table S1). This head defect was more intense and lethality was also strikingly increased by injection with 400 pg of *mNanog* (Table S1), although the lethality did not manifest until the neural stage (data not shown). To examine whether the head defect occurred by apoptosis, we carried out terminal deoxynucleotidyl transferase-mediated deoxyuridine-triphosphate nick end-labeling (TUNEL) assays. *mNanog* injection increased the number of apoptosis-positive cells, suggesting that the head defect was due to apoptosis in the head region (Fig. 1H–J). We then observed the AC shapes. Injection of 200 pg of *mNanog* slightly elongated the AC in the absence of Activin A treatment (Fig. 1K, L), but less so with Activin A treatment (Fig. 1M). This elongation was dependent on the dose of injected *mNanog* (Fig. 1O). On the other hand, elongation of AC by Activin A was suppressed by injection with *mNanog* (Fig. 1N, O). Indeed, RT-PCR analysis of stage-18 AC revealed that *mNanog* injection decreased expression of mesoderm genes such as *ms-actin* [19] and *Xbra* [20] in Activin A-treated AC, whereas the expressions of notochord markers, *chd* and *Xnot*, were upregulated (Fig. 1P). Furthermore, injection of 200 pg of *mNanog* mRNA into the ventral hemispheres of 4-cell embryos induced a weak secondary axis formation (Fig. 1Q, R). This induced axis did not include a head structure with eye vesicles (Fig. 1S), suggesting that *mNanog* may function not as a positive regulator of canonical Wnt signaling like *siamois* (*sia*) [21], but instead as a BMP inhibitor like *chd* and *truncated-type BMP receptor* (*tBR*) [5],[22]. Furthermore, HE staining of *mNanog*-injected tadpole revealed both neural structures and notochord (Fig. 1T, U). Together, these results raised the possibility that *mNanog* possesses dorsal mesoderm-inducing activity in *Xenopus* embryo.

### *mNanog* injection promoted expression of dorsal mesodermal genes, but inhibited ventral mesodermal genes in both AC and embryos

**Figure 2:**
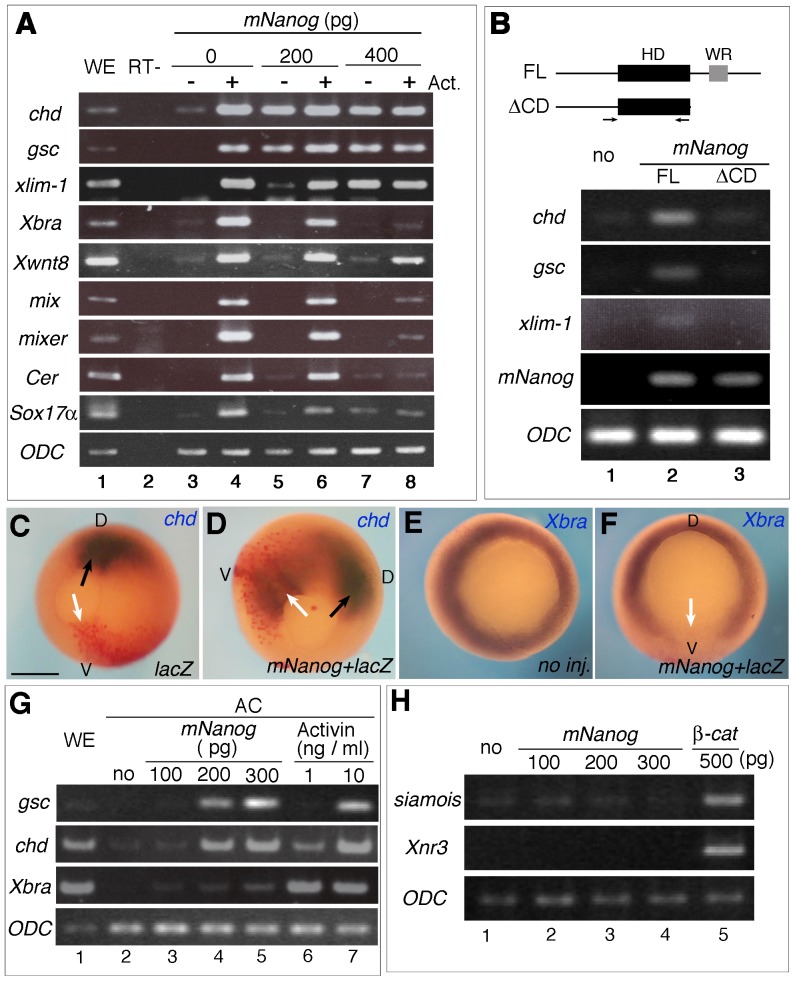
*mNanog* possesses dorsal mesoderm-inducing activity. A) RT-PCR analysis of various marker gene expressions. Expressions of *chd*, *gsc, and xlim-1* (dorsal mesoderm markers), *Xbra* (pan-mesoderm marker), *Xwnt8*, *mix*, *mixer* (ventral mesoderm markers), *Cer*, and *Sox17alpha* (endoderm marker) were observed. *Ornithine decarboxylase* (*ODC*) was also observed as a quantitative control. 200 pg (lane 5, 6) or 400 pg (lane 7, 8) of *mNanog* was injected into the AC region of 2-cell embryos. ACs were dissected at stage 9, treated with 10 ng/ml of Activin A (lane 4, 6, 8), and cultured until stage 11. Whole embryo (WE; stage 11) was also examined. B) Full-length (FL) or a deletion mutant of *mNanog* (deltaCD) was injected and marker gene expressions were observed. Upper column shows a diagram of the *mNanog* construct. Filled and gray boxes indicate the homeodomain (HD) and W-repeat (WR) regions, respectively. Arrow shows the position of primers for RT-PCR. Lower column shows the result. Non-injected AC (lane 1); full-length (FL) *mNanog* injected (lane 2); *mNanog*Δ*CD* injected (lane 3). The level of *mNanog* was also observed to check the precision of injection (4th column). C–F) The effects of *mNanog* injection on endogenous *chd/Xbra* expressions. Scale bar; 500 µm. Expressions of *chd* in stage-12 embryos injected with 800 pg of *lacZ* (C) alone or 200 pg of *mNanog* and 400 pg of *lacZ* (D) into the ventral marginal zone at the 4-cell stage. These patterns are representative of 17/17 (C) and 12/15 (D) embryos. Black arrow indicates endogenous *chd* expression. Expression of *Xbra* in stage-11 embryos injected with nothing (E) or 200 pg of *mNanog* and 400 pg of *lacZ* (F) into the ventral marginal zone at the 4-cell stage. These patterns are representative of 9/9 (E) and 8/11 (F) embryos. White arrow indicates the *mNanog*-injected region. Injected area was active-stained by Red-Gal (Except for (E)). D: Dorsal. V: Ventral. G) Comparison of mesodermal gene expressions between AC cells injected with several doses of *mNanog* (lane 3–5) and those treated with Activin A (lane 6, 7). We observed the expression of *gsc* (1st column), *chd* (2nd column), *Xbra* (3rd column), and *ODC* (4th column). H) *mNanog* did not induce target genes of early canonical Wnt signaling. 100 pg (lane 2), 200 pg (lane 3), or 300 pg (lane 4) of *mNanog* was injected into animal poles and dissected at stage 8. Similarly, 500 pg of *ß-catenin* was injected and dissected (lane 5). Transcription of *siamois* (1st column) and *Xnr3* (2nd column) was observed.

The phenotypes of *mNanog*-injected embryos and their corresponding ACs suggested to us that *mNanog* could induce dorsal mesodermal tissues. We next performed RT-PCR analysis to examine the expression of mesodermal genes in earlier stages. When 200 pg of *mNanog* mRNA was injected into 2-cell embryos, the expression of dorsal mesodermal marker genes *chd*, *gsc*, and *xlim-1* was increased in stage-11 ACs without Activin A treatment (Fig. 2A 1st–3rd column; lane 3, 5), and 400 pg of *mNanog* mRNA injection further increased these gene expressions (Fig. 2A column 1, 2, 3; lane 7). The *mNanog* injections only slightly enhanced the same expressions in Activin A-treated AC (Fig. 2A 1st–3rd column; lane 4, 6, 8). On the other hand, *Xbra* expression was not effectively induced by *mNanog* injection (Fig. 2A 4th column, lane 3, 5, 7), and induction of *Xbra* expression by Activin A treatment was clearly inhibited by *mNanog* (Fig. 2A 4th column, lane 4, 6, 8). Similar inhibition was observed with *Xwnt8*, *mix*, *mixer*, *Cerberus* (*Cer*), and *Sox17α* [23]–[27] (Fig. 2A 5th–8th columns). To assess whether the enhancement of dorsal mesodermal gene expressions was specific for *mNanog* function, we carried out RT-PCR with a deletion mutant of *mNanog* that produces a protein lacking the C-terminus domain including the W-repeat motif (*mNanog*Δ*CD*; Fig. 2B) [28], [29]. Dorsal marker gene expression was not induced by *mNanogΔCD* (Fig. 2B, 1st–3rd columns). Quantitative analysis of the *mNanog* mRNA also suggested that *mNanog* function in mesoderm induction requires dimerization of the mNanog protein (Fig. 2B).

To examine the effect of *mNanog* on endogenous mesodermal gene expressions, we performed *in situ* hybridization. Endogenous *chd* expression was observed in the dorsal lip region (Fig. 2C, black arrow), and only the control *lacZ* injection did not affect *chd* expression (Fig. 2C, white arrow). When *mNanog* was injected into the ventral marginal zone, ectopic *chd* expression was obviously induced (Fig. 2D, white arrow), suggesting that *mNanog* can induce *chd* expression in embryo, confirming the RT-PCR analysis. *Xbra* expression was seen around the yolk plug in normal embryo (Fig. 2E), but was specifically inhibited in the *mNanog*-injected area (Fig. 2F, white arrow), suggesting that *mNanog* negatively regulates *Xbra* expression. These data also indicated that *mNanog* affects the endogenous expression of mesodermal genes in *Xenopus* embryo.

To further profile the mechanism of mesoderm induction driven by *mNanog*, we next compared the expression of mesodermal marker genes between Activin A treatment and *mNanog* injection. AC from normal embryo did not express any mesodermal genes (Fig. 2G, lane 2), but following treatment with Activin A at the dose of 1–10 ng/ml, *chd* and *gsc* were expressed in a dose-dependent manner (Fig. 2G, lane 6–7). *Xbra* was also efficiently expressed following both 1 ng/ml and 10 ng/ml Activin A treatment (Fig. 2G, lane 6–7). When *mNanog* was injected, *gsc* and *chd* expressions gradually increased (Fig. 2G, lane 3–5), as did *Xbra* expression, although the effect of *mNanog* injection on *Xbra* expression was less enhanced than that induced by Activin A treatment (Fig. 2G, 3rd column).

Several mesodermal genes including *chd* are induced by overexpression of canonical Wnt signaling and *Xnr* genes [5], [30]. We therefore examined the expression of early canonical Wnt signaling target genes in our system. There was no increased expression of *sia* and *Xnr3*, known targets of canonical Wnt signaling, despite the injection of *mNanog* mRNA [21], [31] (Fig. 2H). This result suggested that *mNanog* does not affect canonical Wnt signaling in the embryos stages we examined.

### *mNanog* subsidiary utilizes Activin-nodal signaling for dorsal-mesoderm induction

**Figure 3:**
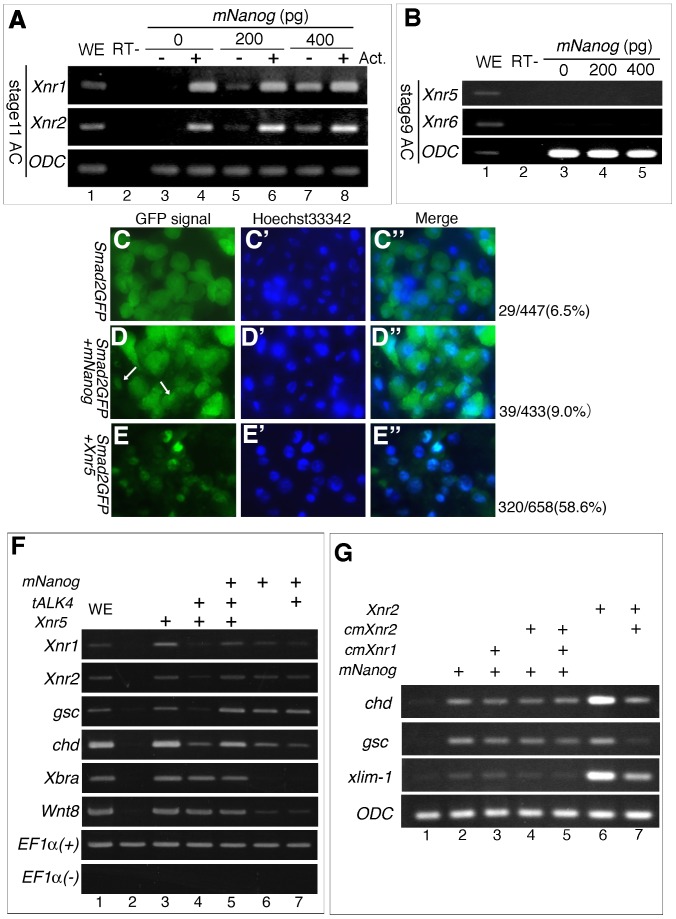
Inhibition of nodal signaling did not effectively reduce dorsal mesoderm gene expression induction by *mNanog*. A) *Xnr1* and *Xnr2* expressions were observed in stage-11 ACs injected with 0 pg (lane 3, 4), 200 pg (lane 5, 6), or 400 pg (lane 7, 8) of *mNanog*, and then treated with Activin A (lane 4, 6, 8). B) *Xnr5* and *Xnr6* expression were observed in stage-9 ACs injected with 0 pg (lane 3), 200 pg (lane 4), or 400 pg of *mNanog*. C–E) Change in intracellular localization of Smad2 with *mNanog* injection. 1 ng of *Smad2GFP* was coinjected with *mNanog* into the animal pole region of 2-cell embryos. AC was dissected from the injected embryos at stage 9 and observed. *Smad2GFP*-injected AC (C, C′, and C″), *Smad2GFP* and 400 pg of *mNanog* injected AC (D, D′, and D″), *Smad2GFP* and 10 pg of *Xnr5* injected AC (E, E′, and E″). For nuclear staining in living cells, Hoechst 33342 was used (C′, D′, and E′). The number indicates cells in which GFP signal was detected in nuclei and total GFP-positive cells. Merged images (C″, D″, and E″). Scale bar: 50 µm. White arrow in (D) indicates nuclear localization of GFP signal with the *mNanog* injection. F) The effect of truncated ALK4 on mesoderm gene induction by *mNanog*. For positive controls, injection with *Xnr5* was also performed. G) The effect of cleavage mutants of *Xnr1* (*cmXnr1*) and *Xnr2* (*cmXnr2*) on mesoderm gene induction by *mNanog*. As a positive control, we used *Xnr2*. In (F) and (G), AC was dissected at stage 9 and cultured to stage 11.

Previously, it was shown that both mesoderm and endoderm formation requires activation of Activin/nodal signaling. Thus, we next examined whether the expression of *Xnr* genes is induced by *mNanog*. RT-PCR analysis indicated that *Xnr1* and *Xnr2* expressions were increased in a dose-dependent manner (Fig. 3A). On the other hand, expression of *Xnr5/6* was not increased in *mNanog*-injected AC (Fig. 3B). From these results, we proposed that mesoderm induction by *mNanog* involves the upregulation of not Xnr5/6, but *Xnr1/2*. To assess whether *mNanog* overexpression promotes the nuclear transport of Smad2, we coinjected embryos with *mNanog* and *Smad2GFP* [32]. Without *mNanog* injection, GFP signal was observed in the cytoplasm of AC cells (Fig. 3C), whereas 10 pg of *Xnr5* injection promoted a nuclear localization of the GFP signal (Fig. 3E). When 200 pg of *mNanog* was coinjected, Smad2GFP signal was occasionally observed in nuclei, although the efficiency was low (Fig. 3D). This result suggested that, at least in some cases, *mNanog* regulates Activin/nodal signaling through *Xnr1/2*.

Next, to clarify whether mesodermal gene induction was dependent on Activin signaling, coinjection experiments were performed with a truncated form of the type I Activin receptor (*tALK4*) [15], which acts as a dominant-negative mutant. Indeed, *tALK4* clearly suppressed *Xnr1, Xnr2, gsc*, and *chd* expressions, but not those of *Xbra* and *xWnt8* (Fig. 3F). When *tALK4* was injected, expression of *Xnr1* and *chd* induced by *mNanog* was slightly suppressed, but *Xnr2* and *gsc* expression was little changed (Fig. 3F, lane 6, 7). We further analyzed the effect of cleavage mutants of *Xnr1* and *Xnr2* (*cmXnr1* and *cmXnr2*) on mesodermal gene induction by *mNanog*. Although *cmXnr1* and/or *cmXnr2* were injected, *chd* expression was only slightly decreased (Fig. 3G, lane 3–5). With *xlim-1*, coinjection with both *cmXnr1* and *cmXnr2* slightly reduced their respective expressions (Fig. 3G, 3rd column, lane 2, 5). Together, these results suggested that *mNanog* weakly modulates Activin/nodal signaling, but that Activin/nodal signaling is not the main factor in mesoderm gene induction by *mNanog*.

### *mNanog* modulated dorsal mesodermal marker genes by regulating BMP signaling via *Xvent2*

**Figure 4:**
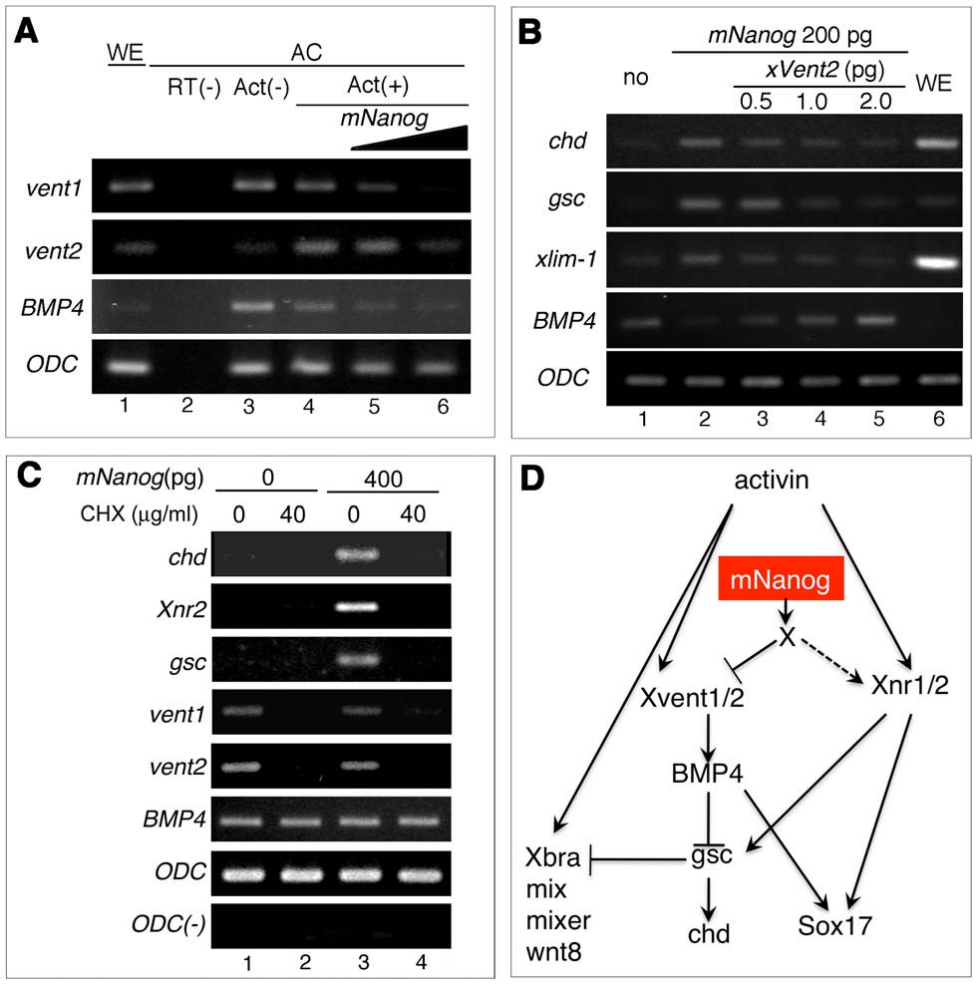
Dorsal mesoderm induction by *mNanog* was involved with inhibition of BMP signaling. A) Target genes of BMP signaling were inhibited by *mNanog* injection, based on the expressions of Xv*ent1* (1st column), *Xvent2* (2nd column), *BMP4* (3rd column), and *ODC* (4th column). 0 pg (lane 3, 4), 200 pg (lane 5), or 400 pg (lane 6) of *mNanog* was injected into animal poles, which were treated with 10 ng/ml of Activin A (lane 4–6) at stage 9. ACs were harvested at stage 11. B) Co-injection analysis with *Xvent2* mRNA. 200 pg of *mNanog* (lane 2–5) and 0 pg (lane 3), 500 pg (lane 4), 1 ng (lane 5), or 2 ng (lane 6) of *Xvent2* were co-injected into animal poles at the 2-cell stage. ACs were dissected at stage 9 and homogenized at stage 11 for RNA preparation. The expressions of several dorsal mesoderm genes (*chd*, *gsc, xlim-1*) and *BMP4* were analyzed. C) Effect of cycloheximide (CHX) on the induction of mesoderm genes by *mNanog*. 0 pg (lane 1, 2) or 400 pg (lane 3, 4) of *mNanog* was injected into animal poles at the 2-cell stage, 0 mg/ml (lane 1, 3) or 40 mg/ml (lane 3, 4) of CHX was added. D) Model of expected mechanism of mesoderm gene induction by *mNanog*. “X" indicates presumptive factor(s) for regulating both *Xvent1/2* and *Xnr1/2* expression by *mNanog*.

We finally examined other marker gene expressions. It is known that dorso-ventral specification in mesodermal tissue involves BMP signaling, and previous reports indicated that *Xvent1* and *Xvent2* facilitate BMP4 transcription, directing ventral mesodermal cell fate [16]. Our results also showed that Activin treatment induced *Xvent1*, *Xvent2*, and *BMP4* expressions in AC (Fig. 4A), and when *mNanog* was injected, these gene expressions were obviously decreased (Fig. 4A, lane 4–6). These results suggested that dorsal mesoderm induction by *mNanog* is dependent on BMP signaling.

Thus, we next examined the effect of coinjecting *mNanog* and *Xvent2*. *Xvent2* and 200 pg of *mNanog* gradually inhibited the expressions of *chd*, *gsc*, and *xlim-1* in a dose-dependent manner (Fig. 4B, 1st–3rd columns). *BMP4* expression was detected in normal AC, and *mNanog* injection inhibited such expression (Fig. 4B, 4th column, lane 2). Interestingly, coinjection of *mNanog* with Xv*ent2* rescued the *BMP4* expression (Fig. 4B, 4th column, lane 3–5), suggesting that *mNanog* suppresses *Xvent2* transcription, resulting in the decreased BMP4 signaling and promotion of dorsal mesodermal gene expression.

To clarify whether *mNanog* function directly or indirectly affects the dorsal mesoderm gene expression, we used CHX treatment to block protein translation. Applying CHX to AC inhibited the expressions of *chd*, *gsc*, and *Xnr2* (Fig. 4C, lane 3, 4), and decreased the expressions of *Xvent1* and *Xvent2* (Fig. 4C, lane 3, 4). These data suggested that both induction of mesoderm genes and inhibition of *Xvent2* expression could be indirectly regulated by *mNanog*.

## Discussion

In this study, we showed a novel function of the *Nanog* gene in *Xenopus* embryo. In the process of LMC analysis with *mNanog*, we found that *mNanog* induces elongation of AC and expression of mesoderm marker genes. Both RT-PCR and *in situ* hybridization showed that *mNanog* effectively induces dorsal mesoderm marker genes, but not ventral mesodermal genes. This is also shown in Fig. 2G as a difference in marker gene induction between *mNanog* injection and Activin A treatment.

On the other hand, elongation of Activin A-treated AC was suppressed by *mNanog* injection (Fig. 1L–M). The expression of ventral mesodermal genes in Activin A -treated AC was inhibited by *mNanog* in both stage-11 and stage-18 embryos (Fig. 1O and Fig. 2A). This inhibitory effect was also observed by *in situ* hybridization for *Xbra* (Fig. 2E, F). Recent study showed *Nanog* functions like x*Vent*, supporting this result [33]. We think that this effect of *mNanog* would be due to upregulation of *chd* and *gsc*, resulting in downregulation of ventral mesoderm gene expression. In the case of Activin A - treated cap or whole embryo under mesoderm-inducing conditions, upregulation of *chd* and *gsc* may drive suppression of the ventral mesoderm gene expression (such as *Xbra, Xwnt8, mix, mixer*) via *gsc* and *chd*. Indeed, as shown in Fig. 2A, dorsal mesoderm gene expression was not decreased by *mNanog* injection. And, untreated AC showed upregulation of several meso/endoderm genes such as *Xwnt8*, *Cer*, and *Sox17α*. In *Zebrafish* embryo, depletion of *Nanog-like* caused inhibition of Sox17expression [34]. Furthermore, it is shown that *Xvent1* could not substitute for *Nanog* function [35]. We think that, in AC cells (without Activin treatment), only upregulation effects could be observed because these ACs have no potential to become ventral mesoderm. In any case, *Nanog* function in mesoderm formation is thought to be complicated, thus further studies need to be done to clarify detail mechanisms.

The *mNanog* injection also caused head defect, and results from the TUNEL assay implicated cell death in the anterior (injected) region as an underlying cause. Injection with 400 pg of *mNanog* induced high lethality in 3-day tadpole (Table S1), confirming the severe effects in *mNanog*-injected regions. We also propose that ectopic expression of a gene possessing mesoderm-inducing activity could affect normal head development. Indeed, 0.25 pg of *Xnr5* injection into animal pole regions caused a similar head defect (data not shown).

In this study, *mNanog* overexpression promoted neither *sia*/*Xnr3* nor *Xnr5*/*Xnr6* expressions (Fig. 2H, 3B), suggesting that *mNanog* could not affect early embryonic signaling such as canonical Wnt signaling and maternal Nodal signaling. On the other hand, both *Xnr1* and *Xnr2* expressions were enhanced by *mNanog* injection (Fig. 3A). The simplest idea to account for these findings is that *mNanog* upregulates *Xnr1/2* transcription, promoting Activin/nodal signaling and *gsc/chd* transcription. However, RT-PCR analysis with *tALK4*, *cmXnr1*, and c*mXnr*2 showed that these dominant-negative genes did not effectively inhibit dorsal mesoderm gene expression (Fig. 3F, G). Nevertheless, *mNanog* actually induced *Xnr2*, and *tALK4* weakly suppressed *Xnr1* and *chd* expression, thus it is suggested that *mNanog*, at least partially, modulates Xnr signaling and contributes to dorsal mesoderm gene induction.

In Fig. 4, we showed that dorsal mesoderm induction by *mNanog* is closely involved with inhibition of BMP signaling. Indeed, *mNanog* injection inhibited *Xvent1*, *Xvent2*, and *BMP4* gene expressions (Fig. 4A), and coinjection of *mNanog* with *Xvent2* clearly suppressed *chd*, *gsc*, and *xlim-1* expression (Fig. 4B). Together with the CHX experiment, our data implicated the dorsal mesoderm-inducing activities of *mNanog* in the modulation of BMP signaling, possibly by indirectly regulating *Xvent*1/2 expression. Our results can be used to propose a model for the modulation and induction of mesoderm genes (Fig. 4D) In short, *mNanog* positively regulates *Xnr2*, but it inhibits expression of BMP factors such as *Xvent1/2* and *BMP4*, resulting in induction of *chd* and *gsc*. This function is similar to that of *Tsukushi* (*TSK*), which modulates both nodal and BMP signaling [36], suggesting that *mNanog* might be involved with the regulation of *TSK*.

Even though our experiments were conducted in an artificial system, we think they are still important in clarifying a novel mechanism involving *mNanog* function, as well as suggesting a novel means of endogenous mesodermal induction in *Xenopus*. This proposed *mNanog* function of mesoderm induction in itself seems opposite to its role in maintaining the undifferentiated state. However, *Nanog* is a possible target gene of Activin signaling [37], [38], and low doses of Activin A are important in maintaining the pluripotency of ES cells in some conditions [39], [40]. Although our results indicated involvement of *mNanog* in Activin/nodal signaling, they also suggested that *mNanog* contributes, at least in part, to the gene regulation mechanism around Activin/nodal signaling that underpins mesoderm formation in *Xenopus*. We expect that other factors involved with pluripotency, like *Oct3/4* and *Sox2*, could also induce activity similar to that observed with *mNanog*, although our preliminary findings showed no mesoderm gene induction following coinjection with x*Sox2* or *Oct61* (data not shown).

This study sought to identify the *Xenopus* gene homolog of mammalian *Nanog* by using sequences of axolotl and newt [41], [42]. Although we designed six primers in homeodomain and caspase domain (Fig. S1 and M&M section) and performed seven rounds of degenerate PCR using combination of these primers, we failed to find any sequence identified as x*Nanog*, although many identified were similar genes including *Xvent1* (6/16) and *Hoxd11* (6/16) (Fig. S1). Moreover, whole genome analysis of *Xenopus tropicalis* revealed no known nucleotide sequence for the *XtNanog* gene. Further exploration of *Xenopus Nanog* or another factor that substitutes for *Nanog* is obviously needed.

## Supporting Information

Figure S1 **Summary of the degenerative PCR for cloning of the** ***Xenopus Nanog*** **gene.** Upper panel: schematic diagram of Nanog protein. CD, HD, and WR indicate the caspase domain, homeodomain, and tryptophan-rich domain, respectively. U1—2 and L1–4 indicate primer positions for the PCR. Lower panel: summary of degenerative PCR results. In Ex.6, we performed PCR with an amplified product using the U2 and L1 primers as a template. The number of obtained gene fragments is also shown.
(TIF)

Table S1 **The summary of phenotypes in embryos injected with** ***mNanog*** **into AP region.**
(DOCX)
