# Supplementary figures and images for: mNanog Possesses Dorsal Mesoderm-Inducing Ability by Modulating Both BMP and Activin/Nodal Signaling in Xenopus Ectodermal Cells
Source: PLoS One. 2012 Oct 11;7(10):e46630. doi: 10.1371/journal.pone.0046630 (PMC3469649; doi:10.1371/journal.pone.0046630)

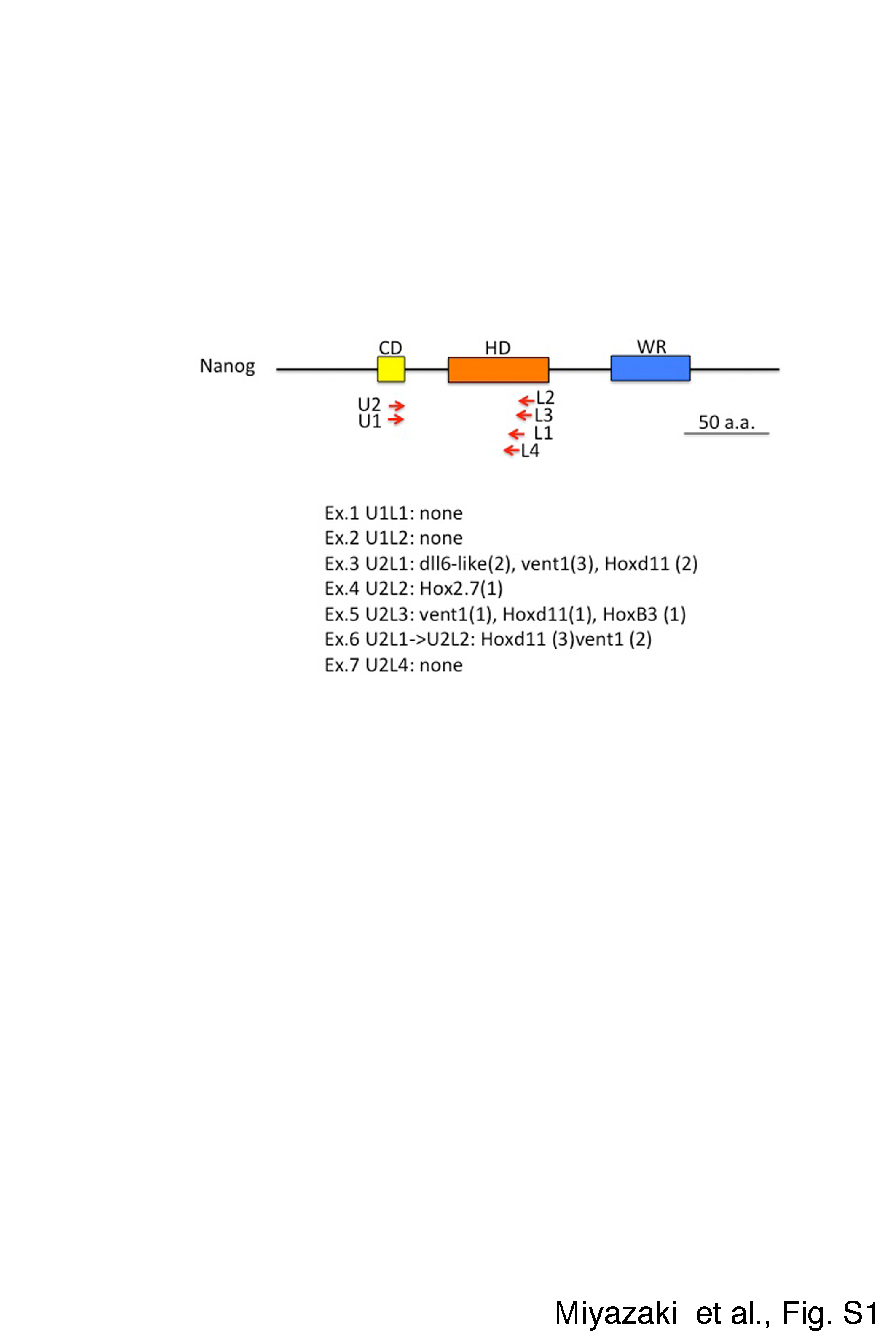

Supplement: Figure S1 — Summary of the degenerative PCR for cloning of the Xenopus Nanog gene. Upper panel: schematic diagram of Nanog protein. CD, HD, and WR indicate the caspase domain, homeodomain, and tryptophan-rich domain, respectively. U1—2 and L1–4 indicate primer positions for the PCR. Lower panel: summary of degenerative PCR results. In Ex.6, we performed PCR with an amplified product using the U2 and L1 primers as a template. The number of obtained gene fragments is also shown. (TIF) [file pone.0046630.s001.tif]
