# Supplementary material for: mNanog Possesses Dorsal Mesoderm-Inducing Ability by Modulating Both BMP and Activin/Nodal Signaling in Xenopus Ectodermal Cells
Source: PLoS One. 2012 Oct 11;7(10):e46630. doi: 10.1371/journal.pone.0046630 (PMC3469649; doi:10.1371/journal.pone.0046630)
Supplement: Table S1 — The summary of phenotypes in embryos injected with mNanog into AP region. (DOCX) [file pone.0046630.s002.docx]

Table S1. The summary of phenotypes in embryos injected with *mNanog* into AP region.

head defect

normal weak strong bent axis dead total

no injection 136 0 0 0 4 140

*mNanog* 100 pg 32 22 3 0 5 62

200 pg 4 41 32 16 19 112

400 pg 2 0 3 1 192 198
